# Identification of MiR-21-5p as a Functional Regulator of Mesothelin Expression Using MicroRNA Capture Affinity Coupled with Next Generation Sequencing

**DOI:** 10.1371/journal.pone.0170999

**Published:** 2017-01-26

**Authors:** Chiara De Santi, Sebastian Vencken, Jonathon Blake, Bettina Haase, Vladimir Benes, Federica Gemignani, Stefano Landi, Catherine M. Greene

**Affiliations:** 1 Respiratory Research Division, Department of Medicine, Royal College of Surgeons in Ireland, Dublin, Republic of Ireland; 2 Genomics Core Facility, EMBL European Molecular Biology Laboratory, Heidelberg, Germany; 3 Department of Biology, University of Pisa, Pisa, Italy; IRCCS-Policlinico San Donato, ITALY

## Abstract

MicroRNAs (miRNAs) are small non-coding RNAs that regulate mRNA expression mainly by silencing target transcripts via binding to miRNA recognition elements (MREs) in the 3’untranslated region (3’UTR). The identification of *bona fide* targets is challenging for researchers working on the functional aspect of miRNAs. Recently, we developed a method (miR-CATCH) based on biotinylated DNA antisense oligonucleotides that capture the mRNA of interest and facilitates the characterisation of miRNAs::mRNA interactions in a physiological cellular context. Here, the miR-CATCH technique was applied to the mesothelin (*MSLN*) gene and coupled with next generation sequencing (NGS), to identify miRNAs that regulate *MSLN* mRNA and that may be responsible for its increased protein levels found in malignant pleural mesothelioma (MPM). Biotinylated MSLN oligos were employed to isolate miRNA::*MSLN* mRNA complexes from a normal cell line (Met-5A) which expresses low levels of MSLN. MiRNAs targeting the *MSLN* mRNA were identified by NGS and miR-21-5p and miR-100-5p were selected for further validation analyses. MiR-21-5p was shown to be able to modulate MSLN expression in miRNA mimic experiments in a panel of malignant and non-malignant cell lines. Further miRNA inhibitor experiments and luciferase assays in Mero-14 cells validated miR-21-5p as a true regulator of *MSLN*. Moreover, *in vitro* experiments showed that treatment with miR-21-5p mimic reduced proliferation of MPM cell lines. Altogether, this work shows that the miR-CATCH technique, coupled with NGS and *in vitro* validation, represents a reliable method to identify native miRNA::mRNA interactions. MiR-21-5p is suggested as novel regulator of *MSLN* with a possible functional role in cellular growth.

## Introduction

MicroRNAs (miRNAs) are small non-coding RNA molecules, 20–25 nucleotides long, highly conserved in the plant and animal world. They play a major role in post-transcriptional regulation, mainly silencing target mRNAs by binding to miRNA recognition elements (MREs) in the 3’untranslated region (3’UTR) and thus decreasing their corresponding protein levels. Although the basic mechanisms underlying their biogenesis and function are mostly known, the identification of *bona fide* targets of miRNAs represents the most challenging aspect for researchers in this field. Recently, different methods have been developed to identify multiple miRNAs binding to a single mRNA of interest [1,2]. Since these methods are limited to the 3’UTR of the mRNA, miRNAs targeting the 5’UTR or the coding sequence cannot be identified.

More recently, we have developed an alternative experimental approach using biotin-conjugated oligonucleotides and magnetic beads [3]. This protocol provides a reliable method to identify specific mRNA::miRNA interactions in a cellular context that decreases the chance of false-positive results compared to miRNA over-expression methods [4]. Moreover, miRNA identification is not limited to the 3’UTR, since mRNAs are studied in their physiological environment, thus miRNAs binding to MREs in the 5’UTR and coding sequence can also be captured. In the original report miRNAs binding the mRNA of interest were identified by using qRT-PCR or miRNA profiling [3]. Recently, this technique was employed to show the physical binding between miR-659-3p and progranulin (*GRN*) mRNA in neuroblastoma cell lines [5]. Moreover it has also been used to validate novel miRNA regulators of *Rac1* mRNA in mouse retina (A. Palfi, personal communication). Here we employ next generation sequencing (NGS) for downstream analysis thereby offering a more unbiased and accurate method to characterise the miRNAs binding the captured mRNA.

In the present work, the “miR-CATCH” technique, as it was then named [6], was further developed and applied to discover miRNAs regulating the human *Mesothelin* (*MSLN*) gene. *MSLN* encodes a ~70kDa precursor protein, that is cleaved into a ~ 31kDa soluble protein (megakaryocyte potentiating factor, MPF) and a ~ 40kDa membrane bound glycoprotein (mature mesothelin, MSLN) [7]. Although the physiological role of this protein is still unknown [8], over-expression of MSLN is often observed in some types of human tumours, namely MPM (Malignant Pleural Mesothelioma), pancreatic and ovarian carcinomas [9]. Moreover, it has been reported that MSLN could have a driving role in cancer by regulating cell proliferation and invasiveness [10,11]. The mechanisms of MSLN over-expression in cancer have not yet been elucidated. Hypomethylation of the *MSLN* promoter and transcriptional up-regulation have been suggested in MPM [11,12], pancreatic [13,14] and ovarian carcinoma [15,16], but few studies have reported post-transcriptional regulation of *MSLN* by miRNAs [17,18]. The present work aimed to identify previously unreported miRNAs involved in the modulation of MSLN expression. Thus, we first applied the miR-CATCH protocol in the Mero-14 MPM cell line [19], a model of MSLN over-expression [11] that allowed the identification of the best capturing oligo. Then we employed the Met-5A cell line [20] for the miR-CATCH analysis followed by NGS. Met-5A is a mesothelial cell line immortalised with SV40 that expresses low levels of MSLN [11]; given that miRNAs and their targets usually display reciprocal expression patterns, Met5A should therefore express high levels of MSLN-regulating miRNAs. Finally, we validated the role of the miRNAs selected after the NGS in a panel of mesothelial cell lines, and demonstrated a functional role for miR-21-5p in cell proliferation.

## Materials and Methods

### Cell lines

Cell lines were grown in 5% CO_2_ at 37°C in media supplemented with 10% FBS and 1% pen/strep. Non-malignant transformed human pleural mesothelial cells (Met-5A) were purchased from ATTC (American Type Tissue Collection) and cultured in Medium 199 (Gibco in Life Technologies, Cat # 22340–020). Human epithelioid malignant mesothelioma cells (Mero-14) were kindly donated by Istituto Tumori of Genova (National Research Council, Genova, Italy), and maintained in Dulbecco's Modified Eagle Medium (Lonza, Cat # BE12-604F). Human biphasic (MSTO-211H) and sarcomatoid (H2052) malignant mesothelioma cells were kindly donated by the Department of Molecular Medicine in RCSI (Royal College of Surgeons in Ireland, Dublin, Ireland) and maintained in RPMI medium (Lonza, Cat # BE12-702F/U1).

### *MSLN* mRNA::miRNA complex isolation

The following protocol was initially applied to identify the best MSLN capture oligo, and in this process Mero-14 cells were used, since they express a high level of the target mRNA. Once the oligo was chosen, three biological replicates of Met-5A were processed according to the protocol, checked for *MSLN* mRNA enrichment and used in the NGS pipeline to identify one or more miRNAs potentially binding to *MSLN* mRNA.

#### MSLN oligo design

mFold (http://mfold.rna.albany.edu/?q=mfold/RNA-Folding-Form) and UGENE [21] software were employed to visualise the secondary structures of the *MSLN* mRNA (transcript variant 1 NM_005823.5) and to identify suitable sequences for oligo design. Three DNA antisense oligos (MSLN_1, MSLN_2 and MSLN_3) were designed to target ss regions in the *MSLN* secondary structure. The thermodynamic properties of the oligos and their specific complementarity towards *MSLN* mRNA were assessed with Oligo Analyzer software (http://eu.idtdna.com/calc/analyzer) and BLAST (Basic Local Alignment Search Tool, http://blast.ncbi.nlm.nih.gov/Blast.cgi), respectively. A not-targeting scrambled oligo was employed to assess the specificity of the *MSLN* capture: 5’-GTGAGGCGTTGTAAGAGTGGTTAAG-3’. All the oligos were modified with a 3’ biotin-TEG (triethyleneglycol) tail. The main features of the three oligos are reported in Table 1. One long single-strand (ss) region was identified with mFold in the coding sequence between bases 328–358 of transcript variant 1 (NM_005823.5), and another 22 bp ss strand was predicted between bases 1445–1466. These exposed regions were also present in the other transcript variants, respectively between bases 271–301 and 1412–1433 for transcript variant 2 (NM_013404.4) and between bases 267–297 and 1384–1405 for transcript variant 3 (NM_001177355.1). Oligo MSLN_1 was designed within the first ss region, whereas MSLN_2 was designed to bind the full length of the second ss region. MSLN_3 was not complementary to a fully ss region (bases 1619–1643), but mFold software predicted a stretch of 5+3+5 ss bases within this region. Regarding the thermodynamic properties, all the combinations of oligo::target mRNA had a high melting temperature (>70°C) and the self-dimer binding free-energy (ΔG) was less than 15% of the ΔG of the hybridisation oligo::mRNA. The top two off-targets (i.e. mRNAs with the potential to be captured using these oligos) according to BLAST are reported in Table 1 and were tested for non-specific capturing. For MSLN_2, *KRT71* (*keratin 71*, which encodes for a keratin, typical of epithelial tissues) was not expressed in Mero-14. Thus, the third non-specific complementary transcript (*CHMP6*, *charged multivesicular body protein 6*) was included in the off-target analysis of MSLN_2.

**Table 1:** Characteristics of MSLN_1, _2 and _3 oligos.

	MSLN_1	MSLN_2	MSLN_3
**Sequence**	5’-AGAGGCTGGAAATGTTAGGTGGGTT-3’	5’- AGGGTGTCTAGGGTGTCTTTGT-3’	5’-TATTCGGACCCGTTCATGTTCTGGA-3'
**Position**	328–352 bp	1445–1466 bp	1619–1643 bp
**GC**	48%	50%	48%
**Tm oligo::target (0.5 M NaCl)**	70.6°C (ΔG: -48.13 kcal/mole)	77.9°C (ΔG: -39.24 kcal/mole)	72.6°C (ΔG: -48.6 kcal/mole)
**Self-Dimer**	ΔG: -3.14 kcal/mole (6.5%)	ΔG: -4.16 kcal/mole (10.6%)	ΔG: -6.68 kcal/mole (13.7%)
**Sequence complementarity with off-target genes**	18/19 *LRP5L*; 14/14 *NBEA*	15/15 *FBXL16*; 15/15 *KRT71* (14/14 *CHMP6*)	14/14 *CTNND1*; 14/14 *CEP170B*

For each MSLN oligo, the following features are reported: bp sequence, position of complementarity on *MSLN* mRNA sequence (transcript variant 1 NM_005823.5), % GC pairs, melting Temperature (Tm) and binding free-energy (ΔG) of the hybridisation oligo::mRNA, ΔG of self-dimers structures and its % referred to the ΔG of hybridisation with target mRNA, non-specific targets (with bp complementarity) predicted with BLAST. All the thermodynamic properties were calculated with Oligo Analyzer software.

#### miR-CATCH protocol

The miR-CATCH protocol [3] was employed with minor modifications highlighted in the discussion section. Briefly, three confluent 75 cm^2^ flasks of cells were pooled and fixed with formaldehyde (0.2%) to cross-link miR-Ago-RISC complexes with mRNAs, lysed and incubated with MSLN or scrambled biotinylated oligos previously immobilised on streptavidin-coated magnetic beads. An incubation of 60’ tumble—30’ rest—45’ tumble was performed at 37°C to allow the annealing of *MSLN* mRNA to the oligo-beads complexes. After a washing step to minimise the binding of non-specific targets, samples were eluted from the magnetic beads and the formaldehyde cross-links were reversed by proteinase K treatment. The resulting samples were used for RNA extraction (miRNeasy Mini Kit, Qiagen, Cat # 217004), qRT-PCR and next generation sequencing.

#### qRT-PCR for assessment of *MSLN* and off-target mRNAs enrichment

Real-time PCR primers were designed with Primer-Blast and checked for specificity and efficiency (S1 Table). qRT-PCR analyses were performed to evaluate the enrichment of *MSLN* and off-target mRNAs in the MSLN- vs scrambled- captured samples. The enrichment of mRNAs was calculated with the 2^-ΔΔCt^ method using *RPLP0* as a reference gene [22], as a representative of highly expressed mRNAs that might non-specifically bind to the oligo beads.

### Next generation sequencing

Library construction was performed using the NEBNext® Multiplex Small RNA Library Prep Set for Illumina® (Set 1) according to the protocol’s recommendation for low input samples starting with half of the available material as input. For the size selection of amplified cDNA libraries, PCR products were loaded on an agarose gel (4%) and passed on to gel extraction with the MinElute Gel Extraction Kit (Qiagen, Cat # 28604). Sequencing of the libraries was performed on an Illumina MiSeq Platform using the Miseq Reagent Kit v2 reading a standard flowcell with 50 cycles.

Sequence data were trimmed using the Trimmomatic software application (http://www.usadellab.org/cms/index.php?page=trimmomatic) to remove linker sequences. The trimmed sequences were then aligned to the human genome (build Hg19) using the Bowtie aligner [23]. The resulting alignments were resolved to read counts aligning to the mature miRNA coordinates from MiRBase [24] version 20 using custom perl scripting. The detection of differentially expressed miRNAs in MSLN- vs scrambled- captured samples was achieved using DESeq2 Package [25]. Selection criteria for validation analyses of miRNA(s) highlighted by NGS were: (i) a significant enrichment in MSLN- compared to scrambled- captured samples and (ii) a number of reads > 20, indicating a possible biological role of the miRNAs in binding *MSLN*.

### *In silico* prediction of selected miRNAs

The PITA algorithm [26], where the whole length of an mRNA can be tested for miRNA binding, was employed to investigate the predicted binding sites of the selected miRNAs within *MSLN* (transcript variant 1 NM_005823.5) and *CEP170B* (transcript variant 1 NM_001112726.2) mRNAs. Target predictions were not performed on *CTNND1* due to the very low presence of this transcript in the captured samples.

### Experimental validation of miRNAs targeting *MSLN* mRNA through transfection of miRNA mimics

In three independent miRNA mimic experiments, Met-5A, Mero-14, MSTO-211H and H2052 cells were seeded at a density of 250000 cells/well. After 24 hours they were transfected with either miRVana hsa-miR-21-5p or hsa-miR-100-5p mimics (Life Technologies, Cat # MC10206 and MC10188, respectively) at 30 nM, using Ribojuice (Novagen, Cat # 71115–4) in OptiMem-reduced serum media (Life Technologies, Cat # 31985–070). mirVana miRNA Mimic Negative Control #1 (Life Technologies, Cat # 4464058) was used as a negative control. The effect of transfection of miRNA mimics on MSLN protein was assessed at 72 hours after transfection. Briefly, proteins were collected with RIPA buffer (supplemented with Halt Protease inhibitor Cocktail, Cat # 1862209, Thermo Scientific, and 0.5M EDTA solution, Cat # 1861274, Thermo Scientific), resolved by 10% SDS-PAGE, transferred to polyvinylidene difluoride (PVDF) membranes and incubated at 4°C with primary anti-mesothelin antibody (Santa Cruz, Cat # sc-271540, Lot # F0111; overnight, 1:250). Stripping protocol was performed using Restore Western Blot Stripping Buffer (Cat # 21059, Thermo Scientific) on the same membrane to obtain β-actin signal. Briefly, after 15 minutes of incubation with stripping buffer, membranes were washed and treated with blocking solution (3% dry milk, 1% bovine serum albumin). The membranes were then incubated for 30 minutes at 4°C with anti-ß-actin antibody (Millipore, Cat # MAB1501, Lot # 2384641; 30 minutes, 1:50000). Anti-mouse IgG, HRP-linked antibody (Cell Signaling, Cat # 7076S, Lot # 32; 1:2500) was used as a secondary antibody for one hour at RT for both MSLN and β-actin antibodies. Detection was achieved using Immobilon Western Chemiluminescent HRP Substrate (Millipore, Cat # WBKLS0100) and membranes were analysed by densitometry using the ImageLab software. For quantitative analysis, the signal intensity of each band was normalized with ß-actin densitometry values.

In parallel, in order to measure the transfection efficiency, Met-5A, Mero-14, MSTO-211H and H2052 cells were seeded at a density of 50000 cells/well in duplicate in p24-well plates. The transfection protocol with miRVana miRNA mimics was carried out as described above. At 48 hours after transfection, RNA was isolated with Trizol reagent (Sigma-Aldrich, Cat # T9424) and miRNAs levels were measured with TaqMan microRNA Assays (Cat # 4427975, Assay ID 000397 for hsa-miR-21-5p and 000437 for has-miR-100-p) according to manufacturer’s protocol. Reverse transcription was performed with TaqMan® MicroRNA Reverse Transcription Kit (Life Technologies, Cat # 4366596) using stem-loop specific miRNA primers starting from 100 ng of total RNA. Expression of miRNAs relative to U6 snRNA (Cat # 4427975, Assay ID 001973) was determined using the 2^-ΔΔCt^ method [22].

### Experimental validation of miR-21-5p as a regulator of MSLN

MiR-21-5p ability to regulate MSLN was further validated in Mero-14 cells with anti-miR experiments and luciferase assay.

#### Anti-miR-21-5p experiments

In three independent miR inhibitor experiments, Mero-14 were seeded at at a density of 250000 cells/well and then transfected with either miRVana hsa-miR-21-5p inhibitor (Life Technologies, Cat # MH10206) or mirVana™ miRNA Inhibitor Negative Control #1 (Life Technologies, Cat # 4464076) at 100 nM, using Ribojuice (Novagen, Cat # 71115–4) in OptiMem-reduced serum media (Life Technologies, Cat # 31985–070). The effect of transfection of miR-21-5p inhibitor on MSLN protein was assessed at 72 hours after transfection using western blot as previously explained. Routinely the transfection efficiency was measured with qRT-PCR of miR-21-5p levels as previously explained.

#### Luciferase reporter assay

An arbitrarily chosen 223 bp region of the *MSLN* coding sequence (nucleotide position 1051–1273 of the RefSeq NM_005823.5), containing the most likely miR-21 binding site (starting at residue 1237, miRNA seed GATGAGCT), was amplified by Q5® High-Fidelity DNA Polymerase (NEB, Cat # M0491). The resultant PCR amplicon was subsequently cloned downstream to the firefly *luciferase* reporter gene into the XhoI site of the pmirGLO Dual-Luciferase vector (Promega, Cat # E1330) using CloneEZ® PCR Cloning Kit (GenScript, Cat # L00339). This construct is from now on referred as “WT_pmir_MSLN”. Subsequent site-directed mutagenesis reactions were performed with QuikChange Lightning Multi Site-Directed Mutagenesis Kit (Agilent, Cat # 210515), in order to generate a plasmid, referred as “MUT_pmir_MSLN”, with mutations in the predicted binding site (in bold in the miRNA seed GATG**CTT**T). The fidelity of the resulting construct was confirmed by sequencing, using the pmirGLO external primers (pmir_F and pmir_R). The sequence of cloning, mutagenesis and sequencing primers are reported in S2 Table.

In three independent experiments, Mero-14 cells were seeded in 96-wells plates at a final density of 10,000 cells/well and incubated for 24 hours. Cells were then cotransfected at 60–80% confluence with WT/MUT_pmir_MSLN (100 ng) together with, alternatively, miRVana hsa-miR-21-5p mimic or miRVana miRNA Mimic Negative Control #1 (30 nM). Transfections were performed using Genejuice (Novagen, Cat # 70967) from plasmid DNA and Ribojuice for miRNA in OptiMEM reduced serum media as per the recommended conditions. Renilla luciferase expressed within both the WT_ and the MUT_pmir_MSLN reporters was used as an internal control of transfection efficiency. Twenty-four hours after transfection, a Dual-Luciferase Reporter Assay (Promega, Cat # E1910) was performed. Relative luciferase units (RLU) were expressed as mean value of the firefly luciferase/Renilla luciferase ratio of three independent experiments.

### Sulphorhodamine (SRB) and clonogenic assay for functional analyses on miR-21-5p

The effect of miR-21-5p on mesothelioma cell line proliferation was next assessed with SRB assay. In three independent experiments, Mero-14 and MSTO-211H cell lines were seeded in 96 wells plate at a density of 3000 cells/well. The following day, cells were transfected with either miRVana hsa-miR-21-5p or mimic mirVana miRNA Mimic Negative Control #1 at 30 nM, as described previously. In parallel, cells were also seeded and transfected in 6 wells plate for protein extraction as control for transfection. H2052 cells could not be used since they were not able to proliferate after transfection. Mesothelioma cells were tested at 72, 96 and 120 hours post-transfection following the SRB protocol as described elsewhere [11]. At each time point, total proteins were extracted with RIPA buffer and MSLN expression was assessed in western blot analysis as described previously.

The ability of MPM cells to proliferate indefinitely following miR-21-5p transfection was tested by clonogenic assay. In three independent experiments, Mero-14 and MSTO-211H cells were seeded in 6 wells plate at a density of 250000 cells/well. The following day, cells were transfected with either miRVana hsa-miR-21-5p or mimic mirVana miRNA Mimic Negative Control #1 at 100 nM, as described previously. After 24 hours, cells were trypsinised, counted and seed in 6 wells plate at low density (6000–8000 cells/well). Cells were stained with crystal violet after 10 days and colonies (>50 cells) were counted with ImageJ software.

### Statistical analyses

The enrichment of *MSLN* and of the off-target mRNAs in the miR-CATCH pipeline was statistically evaluated with one-way analysis of variance (ANOVA). Dunnett’s multiple comparison tests were performed within the ANOVA model to assess pairwise differences between each group and the control group. For the validation study, the effect of miRNA mimics on MSLN protein levels was evaluated with ANOVA, and the punctual P-values for each comparison were calculated with Dunnett’s test again. The effect of anti-miR-21-5p on MSLN protein levels was evaluated with Student’s *t*-test. For the luciferase assay, the effect of miR-21-5p on the wild type and mutant plasmid was compared to its corresponding negative control with Student’s *t*-test. The influence of miR-21-5p mimic on cell proliferation was assessed with a multifactorial ANOVA (mANOVA), where the time point post-transfection (72, 96 or 120 h) and the treatment (miR-21-5p or negative control mimic) were considered as experimental variables. In the mANOVA, the multiple comparisons were evaluated with Bonferroni’s test. The effect of miR-21-5p transfection on clonogenicity was assessed with Student’s *t*-test. GraphPad Prism 7.0 software package was used for all the statistical analyses.

## Results

### Test for capture efficiency and specificity of MSLN oligos

The miR-CATCH technology uses affinity capture oligonucleotides to co-purify a single mRNA together with all its endogenously bound miRNAs. In this work, *MSLN* mRNA was co-purified with its cognate miRNAs from mesothelial cells pre-treated with formaldehyde, lysed and then incubated with *ad hoc* designed MSLN capture oligos. Three oligos were designed according to RNA structure analyses, thermodynamic properties and specific complementarity to ensure the best capture of *MSLN*.

**Fig 1:**
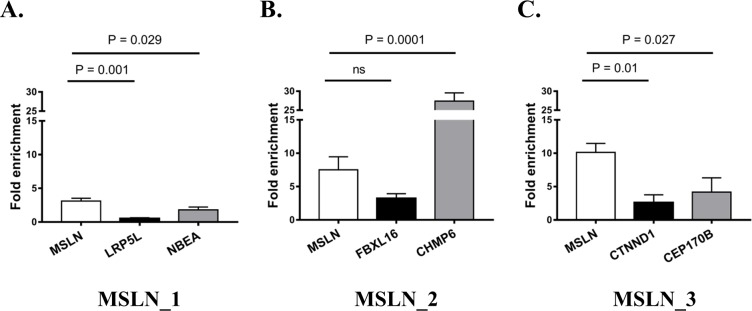
**Test for capture efficiency and specificity of MSLN_1 (A), MSLN_2 (B), and MSLN_3 (C) oligos.** QRT-PCR was used to calculate *MSLN* and off-target mRNA enrichment for each tested oligo in Mero-14 samples. Relative mRNA expression was quantified using the 2^-ΔΔCt^ method comparing MSLN-captured vs scrambled-captured samples. The punctual P-value according to Dunnett’s test calculated within the ANOVA model is also reported. The columns represent mean values, the bars show standard error of the mean (SEM) of three independent experiments. Ns = not statistically significant.

Mero-14 lysates were used (in triplicate) to test the efficiency of *MSLN* capture since their MSLN expression is higher than in non-malignant cells [11]. This allowed the identification of the most effective oligo in a context of high expression of the target gene. For each lysate, a fraction was incubated with MSLN or scrambled oligo, to identify the background noise caused by abundant transcripts that might bind non-specifically to the oligos or to the magnetic beads. The enrichment of *MSLN* and off-target mRNAs was calculated by qRT-PCR comparing MSLN- vs scrambled- captured sample, in the same lysate. Fig 1 shows the fold enrichment of *MSLN* mRNA and off-target mRNA for the three MSLN oligos, expressed as means ± standard error of the mean (SEM). Capture of *MSLN* mRNA with MSLN_1 led to an enrichment of 3.11 (± 0.41), whereas no enrichment (0.53 ± 0.11) was reported for the predicted off-target *LRP5L* (*LDL receptor related protein 5 like*) and only a slight enrichment (1.81 ± 0.23) was evident for *NBEA* (*neurobeachin*) mRNA. Capture with MSLN_2 facilitated better *MSLN* mRNA fold enrichment (7.52 ± 1.94). Regarding the off-target mRNAs potentially captured by MSLN_2, *FBXL16* (*F-box and leucine rich repeat protein 16*) mRNA was lower than *MSLN* (3.23 ± 0.70), but *CHMP6* mRNA was highly enriched (27.40 ± 1.25), thus MSLN_2 was excluded. Capture using MSLN_3 led to the highest *MSLN* mRNA enrichment (10.12 ± 1.36) and the lowest off-target mRNA enrichment (2.61 ± 1.16 for *CTNND1*, *catenin delta 1*, and 4.17 ± 1.24 for *CEP170B*, *centrosomal protein 170B*). Thus, MSLN_3 was chosen to be used in the miR-CATCH protocol for NGS analyses with Met-5A samples, which likely contain less target mRNA [11] but potentially more MSLN-regulating miRNAs.

### *MSLN* mRNA capture for NGS samples

Three independent Met-5A lysates were processed using MSLN_3 oligo. The enrichment of *MSLN* and off-target mRNAs was calculated using the 2^-ΔΔCt^ method [22] comparing MSLN_3- vs scrambled- captured sample. The same samples were then used in the NGS pipeline to identify one or more miRNAs potentially binding to *MSLN*. The fold enrichment of *MSLN* mRNA was significantly higher than the enrichment of the off-target mRNAs (8.95 ± 2.20 for *MSLN* vs 3.10 ± 0.75 for *CTNND1* and 2.57 ± 0.31 for *CEP170B*). Notably, C_T_ values for *CTNND1* were over 35 for all the MSLN- and scrambled- captured RNAs, suggesting very low presence of this transcript in all the samples.

### Next generation sequencing

Analysis of the Illumina short reads identified 325 miRNAs being present in the samples. Raw reads counts for each mature miRNA are reported in S3 Table. Differential Expression analysis performed with DESeq2 in MSLN- vs scrambled- captured samples identified 13 genes with an adjusted p-value of less than 0.1 in Met-5A (S4 Table). Of these, hsa-miR-21-5p (P = 4.4x10^-5^, Fold Change = 2) and hsa-miR-100-5p (P = 0.018, Fold Change = 1.66) passed the criteria to be selected for further validation.

### *In silico* binding prediction of miR-21-5p and miR-100-5p to *MSLN* and off-target mRNAs

The PITA algorithm revealed the presence of four potential binding sites for miR-21-5p within the coding region of *MSLN* mRNA (starting at residues 1237, 1302, 1512 and 1507), among which only the one starting at nucleotide 1237 was predicted to have a negative binding free energy (ΔΔG) (i.e. -5.64 kcal/mol). Since ΔΔG is the energetic score of the annealing process between a miRNA and its target site on the mRNA, the lower (more negative) its value, the stronger the binding of the miRNA is expected to be. Three possible binding sites were predicted for miR-100-5p (one in the 5’UTR and two in the coding region, starting at residues 122, 659 and 910), all with ΔΔG>0. Regarding *CEP170B*, miR-21-5p is predicted to anneal once with a ΔΔG of +8.53 kcal/mol, whereas three putative binding sites are predicted for miR-100-5p, two of whom with ΔΔG<0.

### MiR-21-5p overexpression decreases endogenous levels of MSLN protein in a panel of cell lines

**Fig 2:**
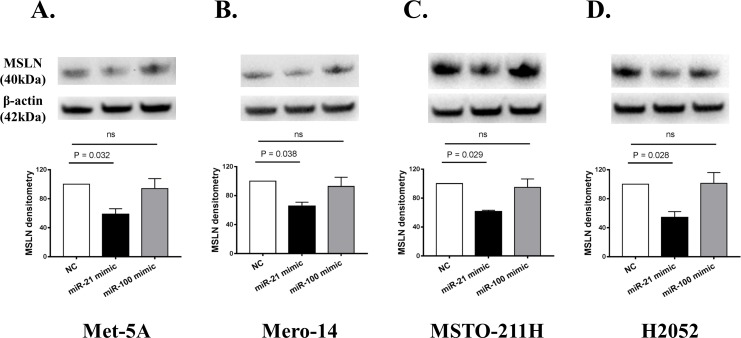
Effects of miR-21-5p and miR-100-5p over-expression in a panel of malignant and non-malignant cell lines. For western blot experiments, quantification after internal normalisation with β-actin is displayed together with a representative image. Met-5A, Mero-14, MSTO-211H and H2052 are shown in A, B, C and D, respectively. Negative control-treated samples are reported as reference and set at 1 (or 100%). The punctual P-value according to Dunnett’s test calculated within the ANOVA model is also reported. The columns represent mean values, the bars show standard error of the mean (SEM) of three independent experiments. Ns = not statistically significant.

As miR-21-5p and, to a lower extent, miR-100-5p were enriched in MSLN-captured samples, their ability to modulate MSLN protein expression was assessed on a panel of non-malignant (Met-5A) and malignant (Mero-14, MSTO-211H and H2052) cell lines with miRNA mimic experiments. As shown in S1 Fig, the levels of miR-21-5p and miR-100-5p in miRNA mimic transfected cells were both significantly higher than those transfected with the negative control, indicating that all the cell lines had been successfully transfected with miR-21-5p and miR-100-5p mimic. A reduction in MSLN protein level was evident after over-expression of miR-21-5p but not of miR-100-5p in all of the cell lines (Fig 2A, 2B, 2C and 2D). After miR-21-5p mimic transfection, MSLN protein levels significantly decreased to 58% (±8%), 65% (±5%), 61% (±2%) and 54% (±8%) of that obtained following transfection with the negative control (set to 100%) in Met-5A, Mero-14, MSTO-211H and H2052, respectively.

### Anti-miR experiments and luciferase assay confirmed that miR-21-5p directly regulates MSLN

**Fig 3:**
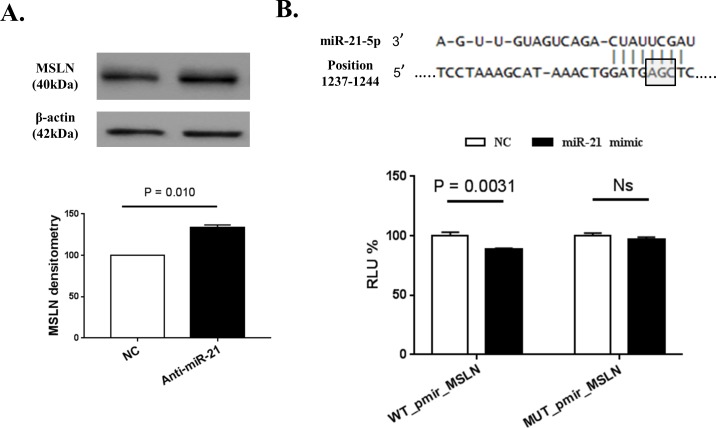
Experimental validation of miR-21-5p as regulator of MSLN expression. Negative control (NC)-treated samples are reported as reference and set at 1 (or 100%). The P-value according to Student’s *t*-test is reported. The columns represent mean values, the bars show standard error of the mean (SEM) of three independent experiments. (A) Effect of miR-21 depletion on MSLN protein in Mero-14 cells. Quantification after internal normalization with β-actin is displayed together with a representative image. (B) Direct interaction between miR-21-5p and *MSLN* is confirmed by luciferase assay in Mero-14 cells. RLU calculated as the ratio firefly luciferase/renilla luciferase are displayed together with the predicted binding site for miR-21-5p on *MSLN* coding sequence. The grey box indicates the three nucleotides (AGC) within the miRNA seed that were mutated to CTT in the MUT_pmir_MSLN plasmid in order to disrupt the miR-21-5p binding site.

In order to validate the role of miR-21-5p as direct regulator of MSLN, we performed anti-miR experiments and luciferase assays on Mero-14 cells. Routinely, after transfection of hsa-miR-21-5p inhibitor, the levels of miR-21-5p were lower (30–50% reduction) than those transfected with the negative control. Following miR-21-5p depletion, MSLN protein levels increased by 33% (±3.4%, P = 0.01) compared to the negative control (set to 100%) (Fig 3A).

Mero-14 cells were transiently transfected with a luciferase reporter plasmid containing the region of *MSLN* coding sequence that most likely is target of miR-21-5p (WT_pmir_MSLN) or a reporter with mutations in the predicted binding site (MUT_pmir_MSLN) to validate the direct interaction between miR-21-5p and *MSLN* suggested by the miR-CATCH. Cotransfection with miR-21-5p mimic resulted in a significant decrease (-13%, ±1.4%, P = 0.0031) of luciferase activity, expressed as RLU, in the WT_pmir_MSLN plasmid, while no significant difference where observed in the MUT_pmir_MSLN plasmid after transfection of miR-21-5p (Fig 3B), demonstrating a direct miRNA-target interaction via the predicted binding site.

### Functional role of miR-21-5p in the regulation of cellular growth

**Fig 4:**
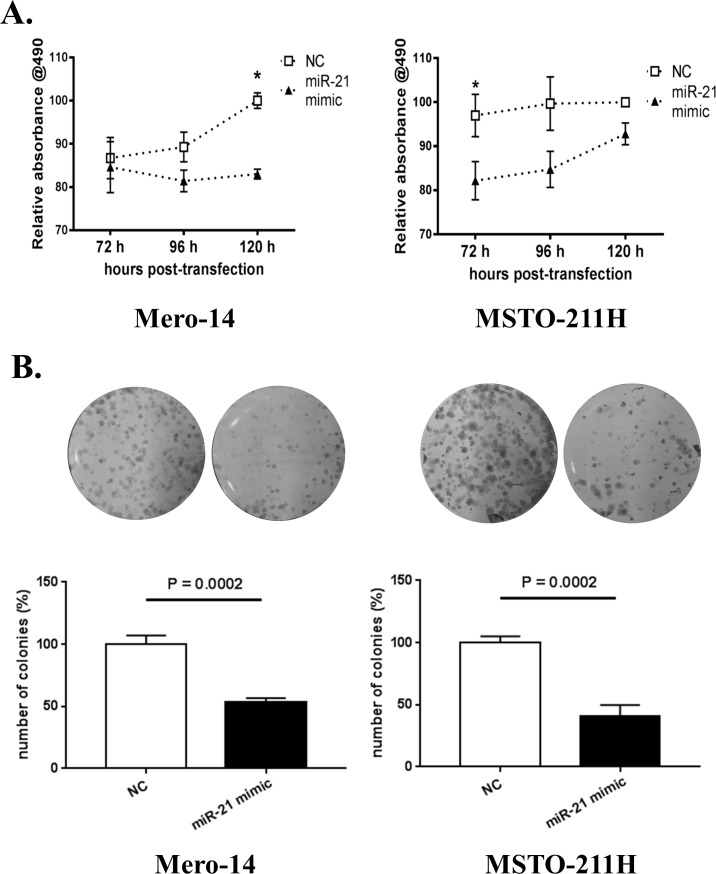
Functional assays on miR-21-5p-transfected MPM cell lines. The columns represent mean values, the bars show SEM of three independent experiments. **(A)** SRB proliferation assay in Mero-14 (left) and MSTO-211H (right) treated with 30 nM of miR-21-5p mimic or negative control mimic. Asterisk (*) indicates P < 0.05 according to Bonferroni multiple comparison test within the mANOVA model. (B) Clonogenic assay: representative image of colonies formed by negative control (NC) or miR-21-5p transfected (100 nM) Mero-14 (left) and MSTO-211H (right) cell lines. The number of colonies is reported in % compared to the average of the three wells transfected with negative control in each experiment. The P-value according to Student’s *t*-test is reported.

The effect of miR-21-5p over-expression on cell proliferation was assessed using an SRB assay at 72, 96 and 120 hours after transfection. S2 Fig shows that treatment with miR-21-5p mimic led to reduction of MSLN protein levels at each tested time point. Compared to the negative control, overexpression of miR-21-5p decreased the rate of proliferation of Mero-14 cells and was 17% lower at 120 h post transfection (Fig 4A, left panel) (P-value = 0.016). In MSTO-211H cells miR-21-5p overexpression also decreased cell proliferation compared to the negative control with the most evident reduction (minus 15%, P = 0.048) at 72 h post transfection (Fig 4A, right panel). Overall, miR-21-5p overexpression led to a significant reduction (P = 0.0069 for Mero-14 and P = 0.0012 for MSTO-211H) in the proliferation rates of both cell lines over time.

To delineate the observed effect on cell growth, we performed clonogenic assays on the same MPM cell lines, whose ability to form colonies was strongly decreased by transfection of miR-21-5p mimic, as compared to control-transfected cells (Fig 4B). The number of colonies in wells treated with miR-21-5p was significantly decreased to 53% and to 40% in Mero-14 and MSTO-211H cells, respectively (P = 0.0002 for both cell lines).

## Discussion

In this work we applied the previously published miR-CATCH protocol [3] to *MSLN* mRNA in order to identify miRNAs targeting this transcript. With this technique, *MSLN* mRNA was successfully isolated using MSLN_3 oligo and its cognate miRNAs were identified by next generation sequencing. *In vitro* experimental analyses validated the findings suggested by NGS, namely that miR-21-5p is a regulator of *MSLN* mRNA.

In the present study we achieved two major goals: the optimisation of the miR-CATCH protocol followed by NGS, and the discovery of a miRNA regulating *MSLN* mRNA that is not predicted with certainty according to the classical prediction algorithms. Regarding the optimisation of the protocol, three important changes in the experimental procedure were introduced: (i) a scrambled oligo was used as a negative control; this represents an improved biological reference compared to the total RNA previously used, since both the MSLN and scrambled oligos underwent the same protocol passages; (ii) a reduced percentage of formaldehyde solution was employed (0.2% instead of 1%), thus preserving RNA quality that can be affected by cross-linking processes [27], and decreasing the background pull-down due to non-specific cross-linking; and (iii) the hybridisation time of miRNA::mRNAs-oligo-beads was extended to 135 minutes (instead of 30 minutes) and performed at 37°C (instead of RT) allowing a better capture and minimising non-specific binding events.

In order to identify novel miRNAs potentially regulating *MSLN* mRNA as reliably as possible, much effort was invested in designing and testing the most effective MSLN oligo in a model of a high concentration of *MSLN* mRNA, i.e. Mero-14 [11]. Although the three tested oligos were predicted *in silico* to have similar thermodynamic properties and to be specific for *MSLN* mRNA, they showed different fold enrichments in their binding with *MSLN* mRNA. Likely, this could be ascribed to different “availability” of the targeted sites in the real cellular environment represented by the Mero-14 cell line. In other words, the capturing process could be affected by unpredictable secondary structures or interaction with other molecules that occur in the physiological context. However, the choice of the oligos was also performed taking into account off-target mRNAs enrichment. Thus, MSLN_2 was excluded due to unacceptably high capture of *CHMP6* mRNA, whereas MSLN_3 was preferable than MSLN_1 because it led to the highest fold enrichment for *MSLN* mRNA with a similar off-target mRNAs capture. Interestingly, one potential off-target, *LRP5L*, which has complementarity of 18 out of 19 bases with oligo MSLN_1, was not enriched suggesting that the presence of a single mismatch in the middle of a predicted binding site can abrogate the binding of the oligo and prevent mRNA capture, confirming the observation we reported previously [3].

The miR-CATCH technique was originally coupled with qRT-PCR or miRNA profiling [3]. Here we demonstrated that the captured miRNAs are amenable to downstream NGS, a more unbiased approach to identify cognate miRNAs. Met-5A cells were used as a model in this approach, since their low levels of *MSLN* mRNA and protein [11] could be ascribed to high presence of targeting miRNAs detectable with NGS. According to our NGS data, the isolation of miR-21-5p and, to a lower extent, of miR-100-5p was significantly higher in MSLN- vs scrambled- captured samples. Experimental validation in a panel of four non-malignant and malignant cell lines using miRNA mimics confirmed miR-21-5p, but not miR-100-5p, as a regulator of MSLN expression. The lack of effect of miR-100-5p could be partially explained by the fact that it is predicted to bind with a stronger affinity to the off-target *CEP170B* rather than to *MSLN* mRNA, as shown by our prediction analysis performed with PITA algorithm. Thus, the possible false positive result for miR-100-5p in NGS may have been due to the presence of *CEP170B* rather than *MSLN* in the MSLN-captured samples.

Further experimental approaches were undertaken in Mero-14 cells in order to validate the role of miR-21-5p as regulator of MSLN expression. The knock down of endogenous miR-21-5p showed a significant increase of MSLN protein levels, further confirming the proposed regulatory mechanism. Since the main feature of the miR-CATCH is the ability to detect physically bound miRNAs to the target mRNA, the direct interaction between miR-21-5p and *MSLN* coding sequence was tested with luciferase assay. Our data showed that miR-21-5p directly regulated MSLN expression via inhibition of luciferase signal from a WT_pmir_MSLN reporter, and that the predicted binding site starting at residue 1237 was indeed functional, as demonstrated by the ineffectiveness of miR-21-5p in decreasing luciferase levels in MUT_pmir_MSLN. These data strengthened the evidence that miR-21-5p has been effectively pulled-down through a direct interaction with *MSLN* mRNA with the miR-CATCH method and that it directly regulates *MSLN*.

Although our major aim was to identify miRNAs regulating MSLN, we also tested the hypothesis that the depletion of MSLN via miR-21-5p transfection could have functional effect on cell proliferation and colony formation ability, as it has been shown previously with siRNA approaches [10,11]. MPM cell lines treated with miR-21-5p mimic showed lower proliferative abilities compared to cells treated with negative controls, both in a classical proliferation assay (SRB) and in the colony formation assay. This could be due to transient depletion of MSLN, which led to a reduction of the cellular growth as already reported by other authors [10,11]. The effects observed on cell proliferation in SRB were modest, but significant. This likely reflects the role of miRNAs as fine-tuners of gene expression. In the clonogenic assay, the decrease of proliferative capabilities was more evident. This could be partially ascribed to different doses of miRNA mimic used in the two assay, but other mechanisms occurring in such a long-term experiment could not be ruled out. For instance, Cioce and collaborators noticed that cell senescence was involved in the clonogenic assay of MPM cell line transfected with miR-145 mimic [28]. The role of miR-21-5p in senescence was beyond the scope of the present study but this and other long-term effects of miR-21-5p could contribute to the more evident response in the clonogenic than in the SRB assay.

Although it has been extensively studied in cancer [29,30,31], miR-21-5p has been poorly investigated in MPM. A role of miR-21-5p in MPM carcinogenesis was reported recently [32,33,34], although other researchers did not observe any differential expression of miR-21-5p between cases and controls [35,36], thus the function of miR-21-5p within MPM biology remains controversial. Whether miR-21-5p expression is altered in other *in vitro* models of MPM or in the mesothelium of patients with MPM *in vivo* is unknown and beyond the scope of the current study. The exact role of miR-21-5p in cancer development and progression is currently unclear and studies on pleural carcinogenesis are lacking. In lung cancer, high expression of miR-21 is associated with poor prognosis [37]. Computational methods suggested that miR-21-5p could play an important role in the development and progression of lung cancer through JAK/STAT, MAPK, Wnt, and PPAR signalling pathways [38]. A number of targets for miR-21-5p have been experimentally validated, including, among the others, *PTEN*, *Bcl2*, *JAG1*, *hMSH2*, *PDCD4* [39,40,41,42,43]. Here miR-21-5p appears to act as a tumour suppressor miRNA since it negatively regulated an oncogene (*MSLN*) [10,11], and this could be explained considering that miRNAs could play different roles depending on the pattern of their target mRNAs expressed in that specific cancer type or tissue.

## Conclusions

In conclusion, this work shows the ability of the miR-CATCH method to generate suitable samples for high-throughput analysis by Next Generation Sequencing. Followed by further experimental validation of miRNA::mRNA interaction, this technique identified miR-21-5p as a regulator of *MSLN* mRNA that may have role in its increased expression and in the proliferation of tumour cells in MPM.

## Supporting Information

S1 Fig Transfection efficiency in non-MPM and MPM cell lines. MiR-21-5p, miR-100-5p or negative control miRNA mimic were transfected in Met-5A, Mero-14, MSTO-211H and H2052 cells, and the transfection efficiency was determined by qRT-PCR. Negative control-treated samples are reported as reference and set at 1. The columns represent mean values, the bars show standard error of the mean (SEM). (A) Relative miR-21-5p levels were significantly higher in the miR-21-5p mimic than negative control transfected cells (the fold change was 3.02±0.46, 8.12±0.21, 14.15±8.71, 34.65±3.95 for Met-5A, Mero-14, MSTO-211H and H2052, respectively); (B) Relative miR-100-5p levels were significantly higher in the miR-100-5p mimic than negative control transfected cells (the fold change was 3.91±0.19, 14.05±0.70, 53.37±4.98, 4.37±0.30 for Met-5A, Mero-14, MSTO-211H and H2052, respectively). *P < 0.05, ** P < 0.01, ***P < 0.001 compared to the negative control-transfected group.
(TIF)

S2 Fig **Effects of miR-21-5p over-expression on MSLN protein levels in Mero-14 (A) and MSTO-211H (B) at 72, 96 and 120 hours after transfection**. Negative control-treated samples are reported as reference and set at 1 (or 100%). The dividing black line represents the splice junction between spliced images coming from different blots. Reduction of MSLN protein levels was evident at all the time points for both cell lines. In Mero-14 cells, MSLN protein levels significantly decreased to 69%, 22% and 80% of that obtained following transfection with the negative control (set to 100%) at 72h, 96h or 120h post-transfection, respectively. In MSTO-211H cells, the levels were reduced to 78%, 71% and 47% at 72h, 96h or 120h post-transfection, respectively.
(TIF)

S1 Table Features of real-time PCR primers employed within the miR-CATCH pipeline. For each primer pair, the sequence, the amplicon length in bp, the concentration in PCR mix and the efficiency calculated with serial dilution of cDNA from untreated Mero-14 cells are given. Data for *KRT1* are not available since this gene was not expressed in this cell line.
(DOCX)

S2 Table Primers names and sequences employed to clone WT_pmir_MSLN and mutate it into MUT_pmir_MSLN. According to the manufacturer’s instructions for the CloneEZ PCR cloning kit, cloning primers covered a 15-base sequence add-on at the 5’-end (capital letters, underlined), an optional restriction site in the middle (capital letter, in bold), and the insert-specific sequence at the 3’-end. MSLN_clon_F and MSLN_clon_R primers were designed to amplify the coding region from 1051 bp to 1273 bp of MSLN mRNA (RefSeq NM_005823.5). For the mutagenic primers, the mutant nucleotides are reported in capital letter, bold. The sequencing primers pmir_seq_F and pmir_seq_R were designed on the plasmid sequence and they were employed for post-cloning screening and sequencing check.
(DOCX)

S3 Table Raw miRNA reads counts for each biological replicate employed in the NGS pipeline. Raw reads counts for each mature miRNA are reported for each sample. Scr = samples produced after capture with scrambled oligo; msln = samples produced after capture with MSLN_3 oligo. 1-2-3 are the biological replicates.
(XLSX)

S4 Table Differentially captured miRNAs according to DESeq2 analysis following NGS. Expression analyses results according to DESeq2 analysis in Met-5A samples when MSLN-captures were compared to scrambled-captures. MiRNAs are ordered according to their adjusted p-value.
(XLSX)
