# Supplementary figures and images for: Identification of MiR-21-5p as a Functional Regulator of Mesothelin Expression Using MicroRNA Capture Affinity Coupled with Next Generation Sequencing
Source: PLoS One. 2017 Jan 26;12(1):e0170999. doi: 10.1371/journal.pone.0170999 (PMC5268774; doi:10.1371/journal.pone.0170999)

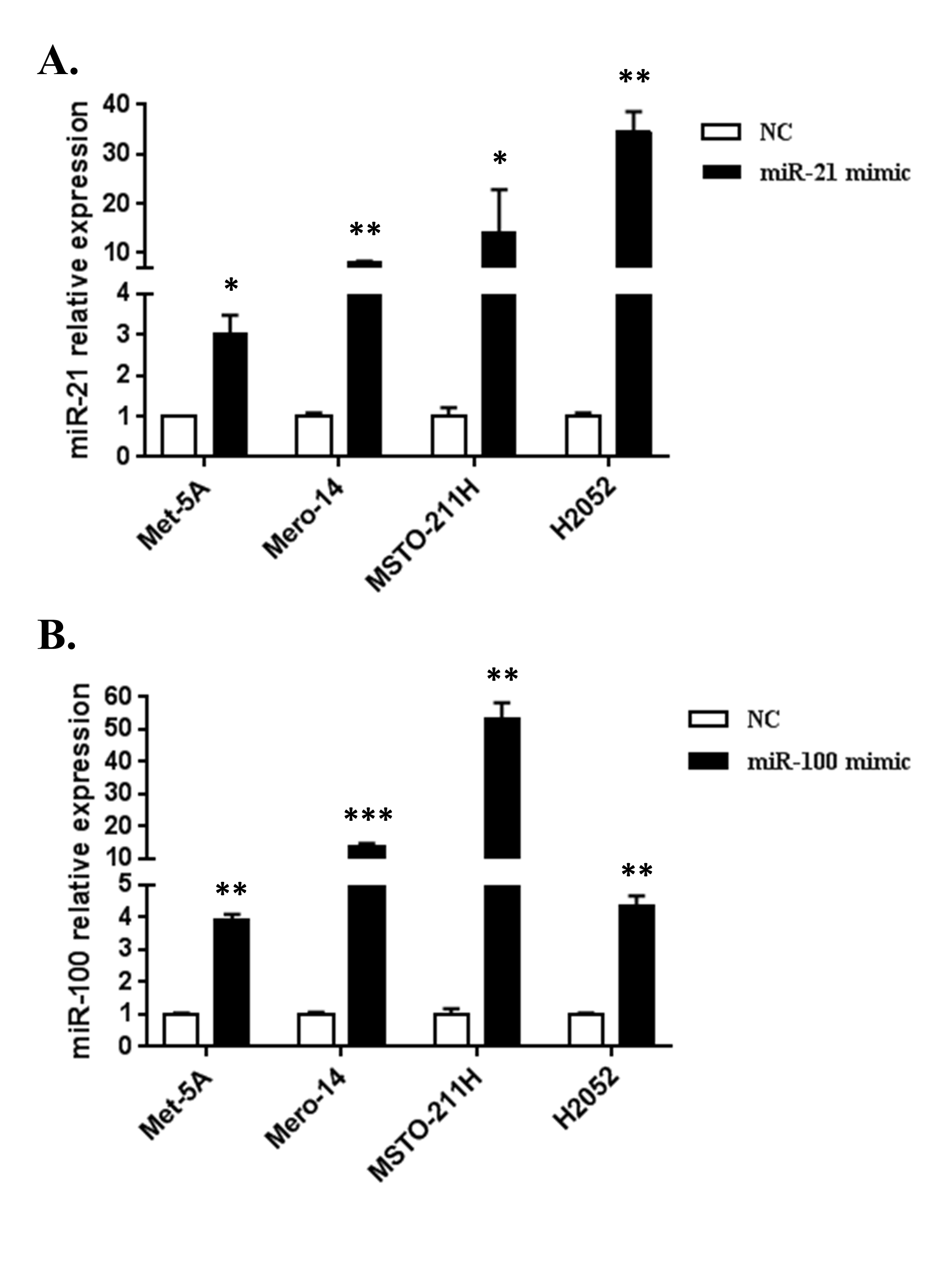

Supplement: S1 Fig — MiR-21-5p, miR-100-5p or negative control miRNA mimic were transfected in Met-5A, Mero-14, MSTO-211H and H2052 cells, and the transfection efficiency was determined by qRT-PCR. Negative control-treated samples are reported as reference and set at 1. The columns represent mean values, the bars show standard error of the mean (SEM). (A) Relative miR-21-5p levels were significantly higher in the miR-21-5p mimic than negative control transfected cells (the fold change was 3.02±0.46, 8.12±0.21, 14.15±8.71, 34.65±3.95 for Met-5A, Mero-14, MSTO-211H and H2052, respectively); (B) Relative miR-100-5p levels were significantly higher in the miR-100-5p mimic than negative control transfected cells (the fold change was 3.91±0.19, 14.05±0.70, 53.37±4.98, 4.37±0.30 for Met-5A, Mero-14, MSTO-211H and H2052, respectively). *P < 0.05, ** P < 0.01, ***P < 0.001 compared to the negative control-transfected group. (TIF) [file pone.0170999.s001.tif]

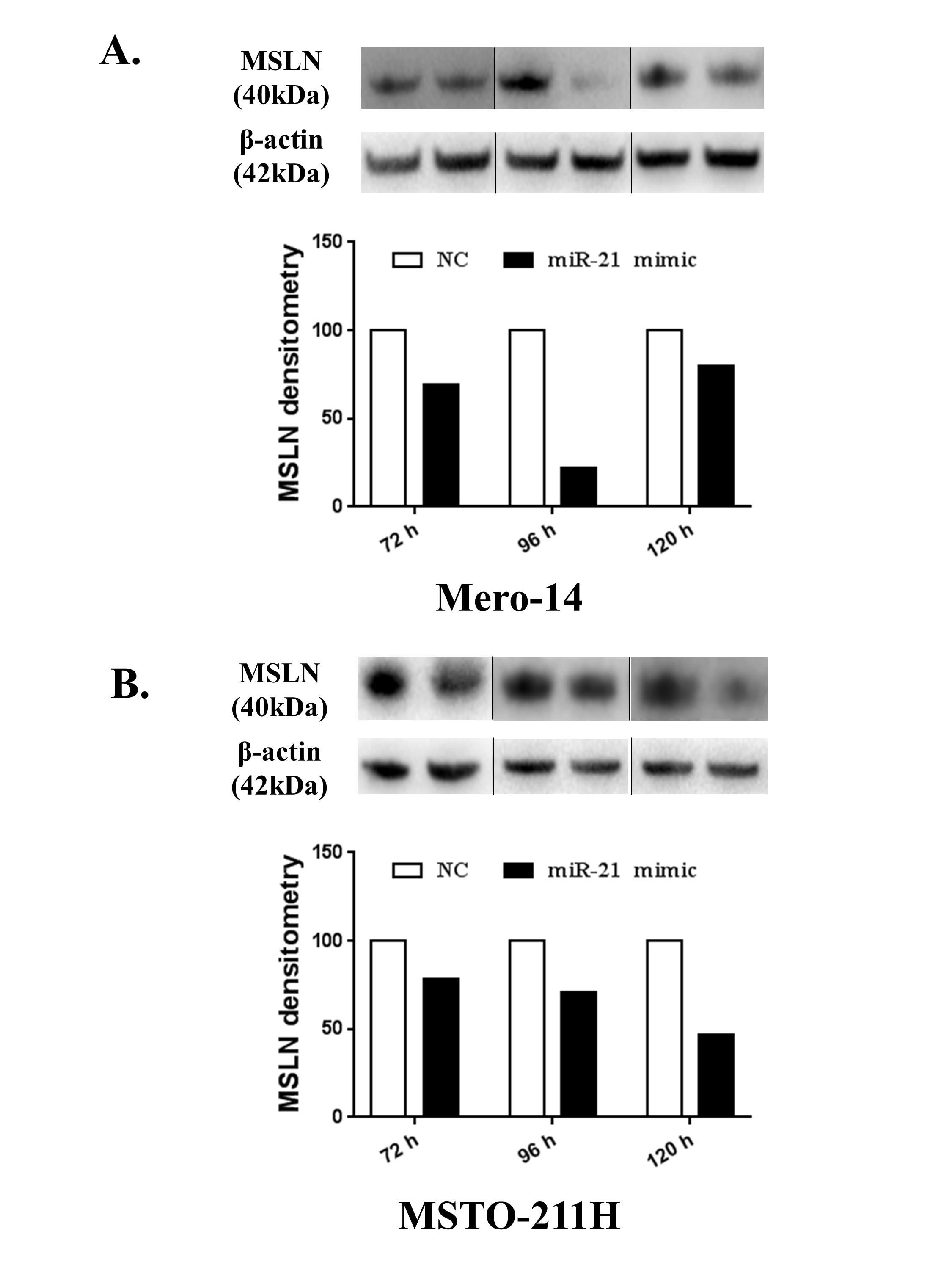

Supplement: S2 Fig — Effects of miR-21-5p over-expression on MSLN protein levels in Mero-14 (A) and MSTO-211H (B) at 72, 96 and 120 hours after transfection. Negative control-treated samples are reported as reference and set at 1 (or 100%). The dividing black line represents the splice junction between spliced images coming from different blots. Reduction of MSLN protein levels was evident at all the time points for both cell lines. In Mero-14 cells, MSLN protein levels significantly decreased to 69%, 22% and 80% of that obtained following transfection with the negative control (set to 100%) at 72h, 96h or 120h post-transfection, respectively. In MSTO-211H cells, the levels were reduced to 78%, 71% and 47% at 72h, 96h or 120h post-transfection, respectively. (TIF) [file pone.0170999.s002.tif]
