# Supplementary material for: Identification of MiR-21-5p as a Functional Regulator of Mesothelin Expression Using MicroRNA Capture Affinity Coupled with Next Generation Sequencing
Source: PLoS One. 2017 Jan 26;12(1):e0170999. doi: 10.1371/journal.pone.0170999 (PMC5268774; doi:10.1371/journal.pone.0170999)
Supplement: S1 Table — For each primer pair, the sequence, the amplicon length in bp, the concentration in PCR mix and the efficiency calculated with serial dilution of cDNA from untreated Mero-14 cells are given. Data for KRT1 are not available since this gene was not expressed in this cell line. (DOCX) [file pone.0170999.s003.docx]

**Supplementary Table 1 (S1)**

|  | **Sequence** | **Amplicon lenght (bp)** | **Concentration (μM)** | **Efficiency** |
| --- | --- | --- | --- | --- |
| ***MSLN*** | F: GCGGGAAGTGGAGAAGACAG  R: TAGGTGAAGGGGATGGCGT | 156 | 0.5 | 97% |
| ***RPLP0*** | F: GGCAGCATCTACAACCCTGA  R: AACATTGCGGACACCCTCC | 81 | 0.5 | 109% |
| ***LRP5L*** | F: CCGAAGCCTCTATTGGACCC  R: CCAGTACGTGAGGCCCATC | 145 | 1 | 105% |
| ***NBEA*** | F: GAAGGCTCTGTGAACCTGGA  R: AAGGAAACACAGGTGCATGGC | 147 | 0.2 | 109% |
| ***FBXL16*** | F: TGCTCGACAGGTGTGTACG  R: AGACAGGAGGCGCAAACTC | 152 | 0.2 | 90% |
| ***KRT71*** | F: TCAGCATCTCCATCATCAGCAG  R: AGGGTGTCTTTGTAATCGTTGG | 172 | N/A | N/A |
| ***CHMP6*** | F: ACCAGCGGCAAATAGACGAG  R: GACAGGGACGTTTTCTGGGA | 158 | 1 | 95% |
| ***CTNND1*** | F: TTGTCTTACGCTCTCTCCTTCC  R: AGAGACCCCCTCACTTCACA | 149 | 0.5 | 100% |
| ***CEP170B*** | F: CAGGTGAGCGTGAAGGGTTT  R: CATACAGGGGTGTGCGGTAA | 145 | 0.2 | 99% |
