# Supplementary material for: Identification of MiR-21-5p as a Functional Regulator of Mesothelin Expression Using MicroRNA Capture Affinity Coupled with Next Generation Sequencing
Source: PLoS One. 2017 Jan 26;12(1):e0170999. doi: 10.1371/journal.pone.0170999 (PMC5268774; doi:10.1371/journal.pone.0170999)
Supplement: S2 Table — According to the manufacturer’s instructions for the CloneEZ PCR cloning kit, cloning primers covered a 15-base sequence add-on at the 5’-end (capital letters, underlined), an optional restriction site in the middle (capital letter, in bold), and the insert-specific sequence at the 3’-end. MSLN_clon_F and MSLN_clon_R primers were designed to amplify the coding region from 1051 bp to 1273 bp of MSLN mRNA (RefSeq NM_005823.5). For the mutagenic primers, the mutant nucleotides are reported in capital letter, bold. The sequencing primers pmir_seq_F and pmir_seq_R were designed on the plasmid sequence and they were employed for post-cloning screening and sequencing check. (DOCX) [file pone.0170999.s004.docx]

**Supplementary Table 2 (S2)**

| **Name** | **Sequence** |
| --- | --- |
| **Cloning primers** |  |
| MSLN_clon_F | AACGAGCTCGCTAGC**CTCGAG**cgggaagtggagaagacagc |
| MSLN_clon_R | CAGGTCGACTCTAGA**CTCGAG**tcacagactcggggtaacct |
| **Mutagenesis primers** |  |
| MSLN_MUT | tcctaaagcataaactggatg**ctt**tctacccacaaggttaccccg |
| **Sequencing primers** |  |
| pmir_seq_F | gtggtgttgtgttcgtggac |
| pmir_seq_R | cagccaactcagcttccttt |
